# Transition to adult care of young patients with neurofibromatosis type 1 and cognitive deficits: a single-centre study

**DOI:** 10.1186/s13023-022-02356-z

**Published:** 2022-05-21

**Authors:** S. Lausdahl, M. M. Handrup, S. L. Rubak, M. D. Jensen, C. Ejerskov

**Affiliations:** 1 Department of Paediatrics and Adolescent Medicine, Centre for Rare Diseases, Aarhus University Hospital, Palle Juul-Jensens Boulevard 99, 8200 Aarhus N, Denmark; 2 Department of Paediatrics and Adolescent Medicine, Center of Paediatric Pulmonology and Allergology, Aarhus University Hospital, Palle Juul-Jensens Boulevard 103, 8200 Aarhus N, Denmark

**Keywords:** Neurofibromatosis type 1, Transition, Psychiatric comorbidities, Cognitive deficits

## Abstract

**Background:**

The transition of adolescents to adult care is known to be challenging. Studies indicate that patients with a chronic disease and cognitive deficits are at risk of inadequate transition to adult care, which eventually may result in disease deterioration. This study investigated the transition process for patients with neurofibromatosis type 1 (NF1) and discussed whether patients with NF1 and cognitive deficits should receive additional attention in their transitional period.

**Method:**

A self-reported online questionnaire assessing disease severity, cognitive deficits, psychiatric diagnoses as well as transition experiences was completed by patients with NF1 aged 15–25-years. Patients were assigned to a national NF1 expert centre covering the western part of Denmark. Furthermore, a retrospective medical chart review was performed, and data were collected to estimate the prevalence of psychiatric diagnoses.

**Results:**

The questionnaire was completed by 41/103 (39%), median age 20 [range 15; 25] years. Medical chart review was performed in 103 patients, median age 20 [range 15; 25]. Participants reporting the transition as difficult all received special needs education, six reported executive function deficits and three out of seven had a psychiatric diagnosis. Fifteen (37%) questionnaire participants reported a wish for more information about the natural history and the prognosis of NF1. The prevalence of psychiatric diagnoses was 24% in the questionnaire survey and 30% in the medical chart review.

**Conclusion:**

This study suggests a need of additional care for patients with NF1 and cognitive deficits including psychiatric disorders during their transition to adult care. In addition, it suggests a need for more information on and education in long-term prospects and mental health issues for patients with NF1.

## Background

Neurofibromatosis type 1 (NF1) is a rare autosomal dominant disorder with an incidence of one case per 3000 caused by germline pathogenic variants in the NF1 tumour suppressor gene [1]. Patients present clinically with dermal manifestations consisting of café au lait spots, freckling, iris hamartomas and dermal neurofibromas [1, 2]. Severe complications in NF1 include optic gliomas, skeletal abnormalities and plexiform neurofibromas with a life-long risk of developing malignant peripheral nerve sheath tumours [2, 3]. Apart from the physical characteristics, learning disabilities, executive functioning deficits, attention deficit hyperactivity disorder (ADHD), attention deficit disorder (ADD) and autism spectrum disorder (ASD) as well as mood and anxiety disorders are common in children and adolescents with NF1 [4, 5]. The aetiology of ADHD, ADD and ASD in patients with NF1 is not fully understood. It is estimated that up to 60% of patients with NF1 suffer from ADHD, although they rarely meet the criteria for the hyperactivity-type. Patients with NF1 are thus often positioned as a combined type or the inattentive type ADD [4, 6]. It is estimated that as many as 25–40% of patients with NF1 meet the criteria for ASD [4, 7].

As children with chronic diseases approach their teenage years, the transition into adult care starts. Generally, the transition in the healthcare system is a well-described and organised process focused on increasingly making the adolescents capable of managing their own disease [8, 9]. The life expectancy for patients with severe chronic disorders has increased, and a successful transition is thus vital to prevent discontinuity of care and deterioration of health. However, often the transition process is unsuccessful [10–12]. Literature in the field is scarce but suggests that patients with severe chronic conditions and developmental disabilities may need a more dedicated focus in the transition process [10, 12, 13]. An Australian study showed that when patients with NF1 lack information about the long-term prospects of the disease, reproductive issues and the severity of the disease, it may lead to deteriorating health due to discontinuity of care [14].

To the best of our knowledge, transition in teenagers and adolescents with NF1 and cognitive deficits has not been studied previously. The aim of this study was to investigate the needs of young adults with NF1 and cognitive deficits. This study was based on data from a self-reported questionnaire and medical chart reviews to describe the diagnostic spectrum of psychiatric diagnoses such as ADHD, ADD and ASD.

## Methods

In Denmark, patients with NF1 are followed in accordance with national clinical guidelines at one of two specialised departments: Centre for Rare Diseases, Aarhus University Hospital (CRD-AUH) or Centre for Rare Diseases, Rigshospitalet, Copenhagen [15]. The centres have a multidisciplinary approach and monitor patients with NF1 from infancy until old age, regardless of severity of NF1. This study was conducted at Centre for Rare Diseases, Aarhus University Hospital, which up take area is half of Denmark’s population. In total 521 patients with NF1 attend CRD-AUH.

### Questionnaire

Patients aged 15–25 years with NF1 were included from the outpatient clinic at CRD-AUH. Patients were eligible for inclusion if they had a diagnosis according to the NIH criteria, met the age criteria and were registered for routine clinical follow up at CRD-AUH [16]. After designing the questionnaire five patients tested the questionnaire and provided feedback.

Patients were invited by letter to complete an anonymous online questionnaire. One month following the invitation, participants received a reminder of the invitation in order to optimize the participation. The questionnaire was designed so that participants could only proceed to the next question if the current question was answered. This eliminated missing data. The questionnaire was divided into age groups; patients aged 15–17 years completed the questionnaire in the beginning of the transition process and patients aged 18–25 years in their post-transition period. The questionnaire was divided into the following six domains and contained 121 closed or semi-open-ended questions: (1) Background characteristics, (2) Disease severity, (3) Executive functions (e.g., difficulties in concentration, memory, navigating, and planning/completing tasks) and psychiatric diagnoses (patient report of diagnosis by a health professional), (4) Well-being, with focus on sexuality, suicidal ideations, and crime, (5) Expectations or experiences with transition from child to adult in the social service sector and (6) Expectations or experiences with health care transition at CRD-AUH.

### Medical chart review

From the medical chart, we collected data on demographics, NF1 diagnostic criteria, psychiatric diagnoses, and number of follow-up visits including non-appearances. Due to ethical regulations, it was not possible to link individual questionnaire data and medical chart data.

### Data collection and analysis

Study data were collected from the questionnaire as well as medical charts and managed using the REDCap (Research Electronic Data Capture) electronic data capture tool hosted at Department of Clinical Medicine, Aarhus University. REDCap is a secure, web-based software platform designed to support data capture for research, allowing the researcher to handle data and perform automated data exports [17]. Descriptive statistics were performed using counts and proportions for categorical variables and median and ranges for continuous variables.

### Ethics

The study was registered by Central Denmark Region ID-number 1-16-02-188-20. Online questionnaires were completed anonymously. The medical chart review was performed as a quality assurance project within CRD-AUH and in accordance with the Ethics Committee of Denmark, no approval is needed for this type of project.

## Results

### Demographic data

**Fig. 1:**
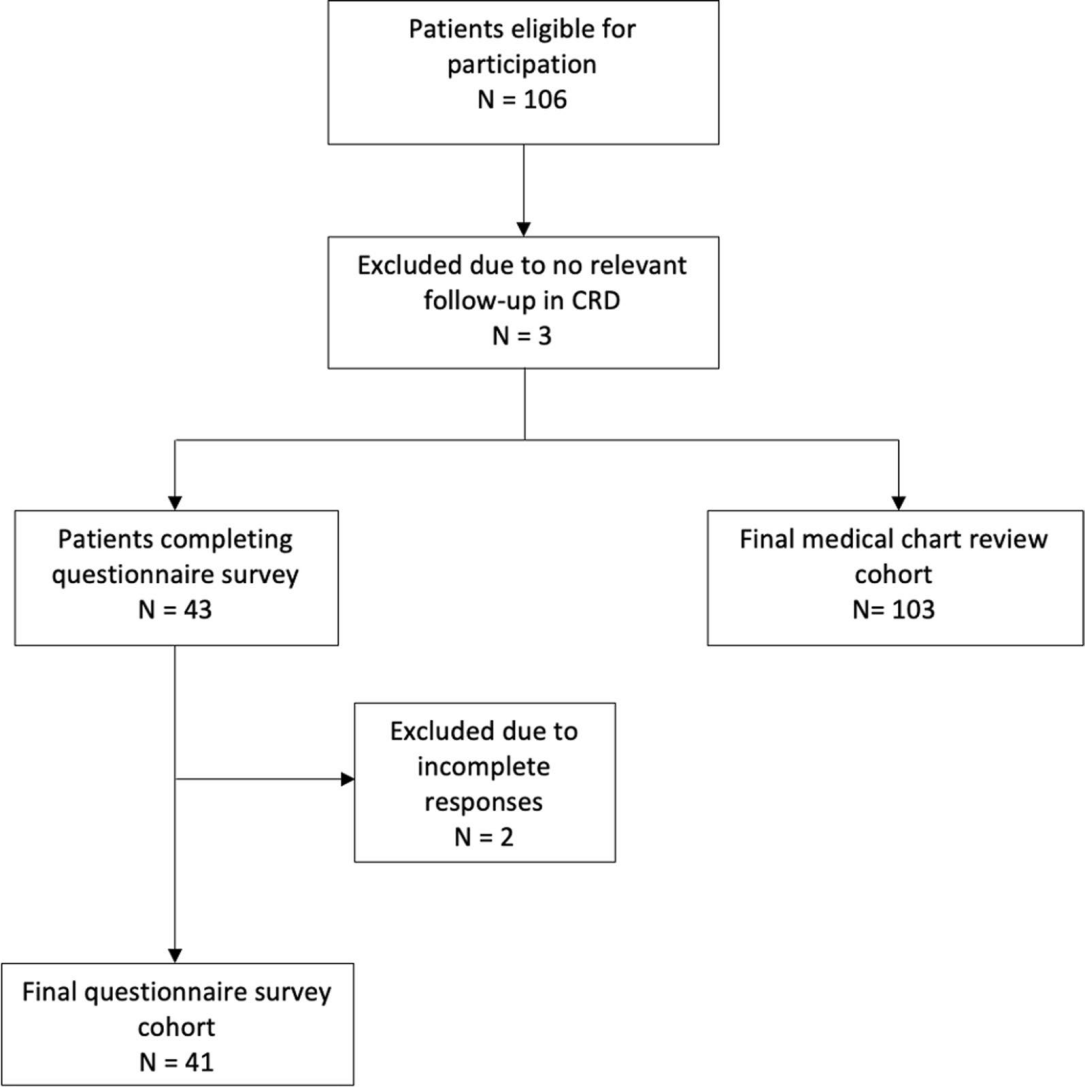
Flowchart of patient inclusion

Figure 1 shows a flow chart of patient inclusion; 103/521 patients were eligible to participate in the study according to inclusion criteria.

A total of 41 completed the questionnaire; ten participants in the group of 15–17-year-olds (4 females, 6 males) and 31 participants in the group of 18–25-year-olds (22 females, 9 males). Thirty-eight (93%) participants had been followed at CRD-AUH since childhood. Five (5/41, 12%) reported they were assisted by a parent to answer the questionnaire due to dyslexia.

The medical chart review was performed in 103 patients (59 males and 44 females) with a median age of 20 years. Twenty-seven (26%) patients were below the age of 18 years (11 females, 16 males). Most participants in both the questionnaire study (85%) and the medical chart review (93%) attended or had completed primary and lower secondary school, which in Denmark ends at ninth grade, at age 15–16 years. However, 60% and 49%, from the questionnaire and medical chart review group, respectively, were dependent on special needs education e.g., extra lessons or a resource teacher, which is an extra teacher who attends to students with cognitive deficits.

Table 1 presents the baseline characteristics of both participants in the questionnaire survey and the medical chart review.

**Table 1:** Baseline characteristics

	Questionnaire, n = 41	Medical chart review, n = 103
Age, median [range]	20 [15;25]	20 [15;25]
Females, *n,* (%)	26 (63)	45 (44)
Mild symptoms (pigmentary changes, neurofibromas, iris hamartomas), *n,* (%)	25 (61)	34 (33)
One or more severe symptoms (PN, opticus glioma, dysplasia), *n,* (%)	16 (41)	69 (67)

*PN* Plexiform neurofibromas

### Psychiatric diagnoses and cognitive function

Table 2 presents psychiatric disorders among patients in both the questionnaire and the medical chart review.

**Table 2:** Psychiatric diagnoses

	Questionnaire, n = 10/41 (24%)	Medical chart review, n = 31/103 (30%)
ADHD, *n,* (%)	1 (10)	13 (42)
ADD, *n,* (%)	2 (20)	4 (13)
Autism, *n,* (%)	0	5 (16)
Depression, *n,* (%)	4 (40)	4 (13)
Bipolar, *n,* (%)	0	1 (3)
Anxiety, *n,* (%)	1 (10)	4 (13)
Eating disorder, *n,* (%)	1 (10)	3 (10)
Other, *n,* (%)	3 (30)	8 (25)

Ten (10/41, 24%) questionnaire participants reported to be diagnosed with a psychiatric disorder; seven had more than one psychiatric diagnosis. The combined prevalence of ADHD, ADD and autism among questionnaire participants was 7% (3/41). Deficits in executive functions were reported by 33 (80%) of the questionnaire participants. Furthermore, 13 (13/41, 32%) participants had experienced suicidal ideations and five (5/41, 12%) were diagnosed with depression or anxiety.

In the medical chart review, the prevalence of psychiatric diagnoses was 31/103 (30%). Eight patients were registered with more than one psychiatric diagnosis. The combined prevalence of ADHD, ADD and autism among patients from the medical chart review was 21% (22/103). Additionally, 15 (15/103, 14%) had milder symptoms of cognitive deficits such as learning disabilities and attention challenges but no psychiatric diagnosis.

### Experiences of transition

#### Experience of health care transition at CRD-AUH

Seven (7/41, 17%) questionnaire participants found the health care transition difficult; three had a psychiatric diagnosis and six reported deficits in executive functions. All seven reported a need for special education.

Fifteen (15/41, 37%) questionnaire participants expressed a wish for more information about the natural history of NF1 and the long-term prospects of the disease. Eight of these received special education, while three had a psychiatric diagnosis. Twelve had deficits in executive functions.

Twenty-two participants (22/41, 53%) reported a wish for more information about NF1 and mental health; four had a psychiatric diagnosis and 13 had a need of special education. Participants reporting the transition as difficult predominately reported a wish for seeing the same doctor for consultations at CRD-AUH.

None of the questionnaire participants reported non-appearance at medical follow-up visits, while in the medical chart review, 14 post-transitional adults were registered with at least one non-appearance during their period of transition; six of these patients were also diagnosed with a psychiatric disorder.

#### Experience of transition in the social service sector

Six (6/31, 19%) post-transitional questionnaire participants found the transition in the sector of social services such as meetings with a social worker and educational counsellor difficult. They predominantly reported it was due to no formalised transition and from the day they turned 18, they had to manage social laws and regulations on their own. Four of these six participants had a psychiatric diagnosis.

All participants were asked if a consultation with a social health care worker employed at CRD-AUH would have been beneficial for their transition; 16 (39%) responded positively to this.

## Discussion

This study is the first in Denmark investigating the needs and experiences in the transition process of patients with NF1. We found that all seven participants (7/41, 17%) who reported the transition to be difficult had cognitive challenges; three had a psychiatric diagnosis, six had executive function deficits and all participants had special educational needs. Our findings support our hypothesis that an additional support during the transition of patients with cognitive deficits would be beneficial. Furthermore, this study is the first in Denmark to describe the prevalence of ADD, ADHD and ASD of 21% (22/103) in adolescents and young adults with NF1. The prevalence of ADHD, ADD and autism in our study was lower than reported in the existing literature, in which a prevalence for ADHD and ADD of up to 60% and of 25–40% for ASD was reported [4, 6, 7]. We noticed that additionally 15 (15/103, 14%) patients in the medical chart review were described with milder symptoms of cognitive deficits such as attention or learning disabilities, which could indicate an underestimation of psychiatric diagnoses in this group. A high proportion (80%) of participants in the questionnaire survey reported deficits in executive functions, which is in line with existing literature [4]. Also, we found a high proportion of patients with a need of special education; 60% in questionnaire and 49% in medical chart review. It seems reasonable to conclude that these challenges may not only affect school and social life, but also make the transition to adult care more difficult. Thus, screening to identify those in a need of additional care during transition should be a part of the transition process. This was also found in a Delphi-study by Fair et al. in which the authors discussed the difference in transition processes for patients with and without developmental disabilities and highlighted the need for a specific transition process adapted to patients with cognitive deficits [13].

Fifteen (15/41, 37%) participants in the questionnaire survey reported a wish for more information about NF1 and the long-term prognosis. When such a need of patient self-management is not fulfilled, the risk of discontinuity of care is more likely, which eventually increases the risk of not detecting disease progression in time [10, 18]. To minimize discontinuity of care, it is highly important to educate the patient in patient self-management and inform them about prognosis and signs of disease progression [8]. Contrary to parents’ expectations, Rietman et al. reported that the need of care increased after the transition [3]. Kennedy et al. [9] found that one of the main issues of an inadequate transition is the risk of non-compliance to treatment, which makes routine clinical attendance less likely. We found that 14 (13%) patients over 18 years had at least one non-appearance at a follow up appointment and 45% of these patients were registered with a psychiatric diagnosis. We do not know the reasons for non-appearances, but the relatively high percentage of non-appearance may indicate the need of more attention during the transition process in patients with NF1 and cognitive deficits.

The questionnaire study also assessed the transition from child to adult in the social service sector context. Transition in the social service sector was difficult due to the abrupt change when patients reached legal age and thus, they have to deal with social laws and regulations on their own. Furthermore, in 39% of the cases, participants were favourably disposed towards a consultation with a social health care worker employed by CRD-AUH. This suggests that a closer corporation between the social system and the health care system would be beneficial, which is in line with a previous study by Reiss et al. [10]. Four out of six participants with an insufficient social sector transition had a psychiatric diagnosis, which underlines the need of additional support for these patients.

In previous studies, mental health is reported to be a major concern for patients with NF1 and their relatives [3, 5]. Twenty-two (54%) participants in the questionnaire survey reported a wish for more knowledge of NF1 and mental health. Surprisingly, we found that 31% reported suicidal ideations and 13% were diagnosed with depression or anxiety. Our study data does not allow us to explore any linkage to NF1. A previous study by Berardelli et al. found suicidal ideation was significantly higher in adults with NF1 compared to the background population and in a recent Danish study, Doser et. al. found a higher prevalence of depression and anxiety among patients with NF1 compared to the background population [5, 19].

The limitations of this study were a low response rate (42%), that more mildly than severely affected patients participated in the questionnaire study and that sixty-three percent of the questionnaire respondents were women, while women constituted 44% of the total cohort. Selection bias should be taken into consideration as patients with psychiatric comorbidity may not be as likely to participate in the questionnaire study. Unfortunately, it was not possible to link the data from questionnaires on patient level to the data from the medical chart review as the questionnaire survey participants were anonymous. Also, selection bias should be considered when generalizing this study, as patients with learning disabilities may be more likely to restrain from answering a questionnaire. Furthermore, patients who went through transition several years ago may experience recall-bias. Our study was not designed with a qualitative methodology. We tried to set up panel discussion with participants in order to discuss the results, which unfortunately was impossible within our time frame, although this would have been relevant. A future study designed as a qualitative interview study including a discussion with the adolescents and young adults with NF1 of this study’s results is now being considered. Also, our study was only performed using retrospective and cross-sectional data, and it was not possible to draw conclusions on the possible outcomes for these patients later in life.

## Conclusion

Our study is among the first to explore the health-care transition process in patients with NF1 and cognitive deficits. The results suggest that patients with NF1 and cognitive deficits may benefit from closer attention in their transition before, during and after the transition process. We found a lower prevalence of ADHD, ADD and ASD, highlighting a probable low recognition of cognitive morbidities among patients with NF1. Our study also highlights the need for better information to the young patient with NF1 on mental challenges and a better education on NF1 disease prognosis and self-management to minimize the risk of discontinuity of care and eventually deterioration in health status of young adults with NF1. This study should act as a pilot study which highlights the need of investigating this topic more thoroughly. A larger qualitative, nation-wide study including interviews or assisted questionnaires is being considered. Also, additional research on prospective outcomes in transition processes is warranted.

## Data Availability

Could be available from the corresponding author on reasonable request.

## List of abbreviations

NF1: Neurofibromatosis type 1
ADHD: Attention deficit hyperactivity disorder
ADD: Attention deficit disorder
ASD: Autism spectrum disorder
CRD: Centre for rare diseases
AUH: Aarhus University Hospital
RedCap: Research electronic data capture

## References

[1] Gutmann DH, Ferner RE, Listernick RH, Korf BR, Wolters PL, Johnson KJ (2017). Neurofibromatosis type 1. Nat Rev Dis Primers.

[2] Friedman JM. Neurofibromatosis 1. In: Adam MP, Ardinger HH, Pagon RA, Wallace SE, Bean LJH, Stephens K, et al., editors. GeneReviews((R)). Seattle (WA) 1993.

[3] Rietman AB, van Helden H, Both PH, Taal W, Legerstee JS, van Staa A (2018). Worries and needs of adults and parents of adults with neurofibromatosis type 1. Am J Med Genet A.

[4] Vogel AC, Gutmann DH, Morris SM (2017). Neurodevelopmental disorders in children with neurofibromatosis type 1. Dev Med Child Neurol.

[5] Doser K, Andersen EW, Kenborg L, Dalton SO, Jepsen JRM, Krøyer A (2020). Clinical characteristics and quality of life, depression, and anxiety in adults with neurofibromatosis type 1: a nationwide study. Am J Med Genet A.

[6] Mautner VF, Kluwe L, Thakker SD, Leark RA (2002). Treatment of ADHD in neurofibromatosis type 1. Dev Med Child Neurol.

[7] Garg S, Green J, Leadbitter K, Emsley R, Lehtonen A, Evans DG (2013). Neurofibromatosis type 1 and autism spectrum disorder. Pediatrics.

[8] Bar C, Ghobeira R, Azzi R, Ville D, Riquet A, Touraine R (2019). Experience of follow-up, quality of life, and transition from pediatric to adult healthcare of patients with tuberous sclerosis complex. Epilepsy Behav.

[9] Kennedy A, Sawyer S (2008). Transition from pediatric to adult services: are we getting it right?. Curr Opin Pediatr.

[10] Reiss JG, Gibson RW, Walker LR (2005). Health care transition: youth, family, and provider perspectives. Pediatrics.

[11] Crowley R, Wolfe I, Lock K, McKee M (2011). Improving the transition between paediatric and adult healthcare: a systematic review. Arch Dis Child.

[12] Van Lierde A, Menni F, Bedeschi MF, Natacci F, Guez S, Vizziello P (2013). Healthcare transition in patients with rare genetic disorders with and without developmental disability: neurofibromatosis 1 and Williams-Beuren syndrome. Am J Med Genet A.

[13] Fair C, Cuttance J, Sharma N, Maslow G, Wiener L, Betz C (2016). International and interdisciplinary identification of health care transition outcomes. JAMA Pediatr.

[14] Oates EC, Payne JM, Foster SL, Clarke NF, North KN (2013). Young Australian adults with NF1 have poor access to health care, high complication rates, and limited disease knowledge. Am J Med Genet A.

[15] National plan for Rare Diseases. https://www.sst.dk/-/media/Udgivelser/2014/National-strategi-for-sj%C3%A6ldne-sygdomme.ashx?la=da&hash=FC2EA0B19FCE0BD6E9DB0391DA2E47F3C6FB03D3: Danish Health Authority; 2014.

[16] National Institutes of health consensus development conference statement: neurofibromatosis. In: Bethesda, Md., USA, July 13–15, 1987. Neurofibromatosis. 1988;1(3):172–8. 3152465

[17] Harris PA, Taylor R, Thielke R, Payne J, Gonzalez N, Conde JG (2009). Research electronic data capture (REDCap)–a metadata-driven methodology and workflow process for providing translational research informatics support. J Biomed Inform.

[18] Ambresin AE, Bennett K, Patton GC, Sanci LA, Sawyer SM (2013). Assessment of youth-friendly health care: a systematic review of indicators drawn from young people's perspectives. J Adolesc Health.

[19] Berardelli I, Maraone A, Belvisi D, Pasquini M, Giustini S, Miraglia E (2021). The importance of suicide risk assessment in patients affected by neurofibromatosis. Int J Psychiatry Clin Pract.

